# Age-related asymmetry in left–right ears of sound lateralization with respect to four different rise times

**DOI:** 10.3389/fnins.2023.1249119

**Published:** 2023-09-05

**Authors:** Kazumoto Morita, Yijie Guo, Takeshi Toi

**Affiliations:** Faculty of Science and Engineering, Chuo University, Tokyo, Japan

**Keywords:** auditory, older adults, aging, interaural time difference, lateralization, brain asymmetry

## Abstract

An experimental investigation was conducted to elucidate the auditory characteristics of the older adult population. The study involved 24 older adult and 24 young participants, with the aim of exploring their horizontal lateralization ability. This was achieved by presenting 1-kHz pure tones to the participants’ right and left ears while introducing interaural time differences (ITDs). We examined the impact of four rise times (2, 5, 20, and 50 ms) on the onset of the test sound. The findings revealed that older adult participants exhibited lower levels of lateralization than young participants. Moreover, both older adult and young participants demonstrated diminished recognition of the onset portion as the rise time increased. Of particular significance was the conspicuous presence of a right ear advantage (REA) among young participants as the rise time was extended (statistically significant between the left and right ears at the 1% level, considering an ITD of 0.8 ms and a rise time of 50 ms). In contrast, older adult participants did not exhibit REA, even with a prolonged rise time (not significant at the 5% level at the same condition). These results indicate that the REA is not only present in language, as previously observed, but also extends to a pure tone in young participants. The older adult participants exhibited reduced performance in both left-and right-ear sound recognition. The influence of hearing threshold and preferred ear on sound lateralization performance was minimal. Therefore, it can be inferred that factors other than hearing threshold or preferred ear contribute to the presence of REA in young participants or its decline with age. The central and/or corpus callosum functions may also contribute to this phenomenon.

## 1. Introduction

In Japan, the proportion of individuals aged 65 years and older has been steadily increasing, and they constitute 29.1% of the total population as of September 2022. This demographic shift is a significant concern not only in Japanese society but also in other developed nations. Older adults need to maintain an active social life; however, functional decline is an inevitable aspect of aging. Regarding cognitive function and decline, in addition to visual dysfunction, auditory impairments such as difficulties in perceiving high-frequency sounds and recognizing speech in noisy environments present notable challenges for this population.

Numerous studies have investigated hearing impairments in the older adult population. Strouse et al. (1998) examined age-related changes in the auditory system by comparing young and older adult participants. Tun et al. (2012) summarized the effect of aging on auditory processing and cognition, revealing that a decline in cognitive abilities precedes reduced engagement in social activities. Eggermont (2017) provided an overview of hearing problems related to deficits in both the auditory periphery and central nervous system, emphasizing the challenge of speech comprehension in noisy social settings, known as cocktail party problems. Anderson et al. (2019) explored age-related cognitive decline and its contribution to difficulties in speech understanding, and proposed novel hearing aid technologies employing neural feedback. Furthermore, Profant et al. (2019) investigated speech perception in silent and naturally noisy environments and examined the interactions between peripheral and central auditory abilities.

We focused on the age-related decline in sound localization ability as a measure of auditory decline (Morita et al., 2019, 2020, 2021, 2023a,b). Sound localization ability has been recognized as a crucial function of auditory information processing, for example, in situations where older adult pedestrians need to determine the direction of approaching emergency vehicles while crossing a road. In our previous studies, we demonstrated the significance of perception of the onset portion in relation to sound lateralization. This aspect of auditory processing has also been recognized by other researchers (Kunov and Abel, 1981; Abel and Kunov, 1983; Balakrishnan and Freyman, 2002; Freyman et al., 2010). These researchers highlighted the dominance of a preceding sound over a succeeding sound in binaural lateralization tasks, and emphasized the concept of onset dominance in lateralization. Buell et al. (1991) documented the efficacy of ongoing delays in quantifying the degree of laterality. Numerous researchers have examined the disparities in the effects between the onset and ongoing portions (e.g., Abel and Kunov, 1983; Freyman et al., 1997), revealing that the extent of such effects can be partly ascribed to the rise time of sound within the experimental conditions.

However, the influence of the onset conditions on sound localization has primarily been investigated in young individuals, with limited studies focusing on older adult participants. Our previous studies (Morita et al., 2019, 2023b) revealed that in the case of a 1-kHz pure tone with a rise/fall time of 2 ms, older adult participants exhibited poorer accuracy in determining the sound direction compared with their younger counterparts when the interaural time difference (ITD) was 0.8 ms. This pattern of performance in the context of a 1-kHz pure tone can be attributed to the phase difference of 288° when the ITD was 0.8 ms, surpassing 180°, resulting in phase reversal. The diminished ability of the older adult participants to accurately perceive the lateralization of sound stems from their inadequate perception of the onset portion, leading them to rely on ongoing cues or make ambiguous judgments. In contrast, young participants demonstrate sufficient recognition of the onset portion (Osawa et al., 2019). For continuous sounds, when the phase exceeds 180°, the opposite ear perceives the sound before the other ear. Hence, the phase angle never exceeds 180°. However, in the case of intermittent or sudden sounds such as alarms, it is crucial to recognize the rising part of the sound for accurate lateralization.

When comparing the auditory characteristics of older adult participants and young participants, it was suggested that conducting experiments under test conditions with slow rise times of the test sound (Morita et al., 2020) or lower sound pressure levels (Morita et al., 2021) might lead to a tendency among older adult participants to confuse the lateralization between the right and left ears. These findings indicate, as aforementioned, that older adult participants exhibited inferior recognition of the onset portion compared with young participants. Furthermore, among the older adult participants, there was occasionally a trend toward poorer performance in lateralizing sounds originating from the right ear, although this was not consistently observed (Morita et al., 2019, 2020).

The objective of this study was to investigate the horizontal lateralization ability of older adults in comparison to that of young adults, examining the impact of rise times on the onset portions. Simultaneously, we aimed to explore potential asymmetries between the left and right ears in these populations. While our research primarily focused on evaluating the lateralization abilities of older adult participants, our overarching aim was to identify their auditory characteristics.

The structure of this paper is as follows. Section 2 details the acoustic experiments conducted with both older adult and young participants. Section 3 presents the study findings regarding hearing thresholds and preferred ear surveys, with specific attention given to lateralization performance outcomes at an ITD of 0.8 ms with respect to four rise times. In section 4, we analyze the discrepancies in lateralization performance between the right and left ears, and discuss the implications of aging on the phenomenon of right ear advantage (REA) in relation to pure tones.

## 2. Materials and methods

### 2.1. Experimental outline

To assess the auditory lateralization ability of both older adult and young participants, the experiment employed a controlled setting wherein the test sounds were delivered individually to each participant via a pair of headphones in a soundproof room.

The test conditions involved presenting sounds at a frequency of 1 kHz and manipulating two variables: the rise time (2, 5, 20, and 50 ms) and the ITD (0.2, 0.4, 0.6, and 0.8 ms, leading either to the right or left ear, as well as the zero ITD condition). This study focused on the influence of rise time as the primary area of investigation.

The participants were instructed to indicate the perceived direction of the sound presented. No visual stimulation or cues were presented to the participants. Trials encompassing all the aforementioned conditions were arranged randomly to form a test block. Each test block consisted of 36 trials, incorporating all four rise times and nine ITDs.

The test block was repeated 10 times for each participant and intervened with breaks. During the latter half of the experiment (i.e., the second five of the 10 blocks), the headphones were reversed from left to right, effectively counterbalancing any remaining asymmetry in the experimental apparatus between the ears.

### 2.2. Test sound and apparatus

Test tones with a frequency of 1 kHz were generated using MATLAB and saved as 16-bit wav files at a sampling rate of 48 kHz. The test tones were administered to each participant using STAX SR-L500 MK2 headphones. The test tones were stored on a computer and converted into analog signals using a TASCAM UH-7000 digital-to-analog converter.

To ensure the absence of extraneous noise during the rising phase, four rise times were established based on preliminary experiments. In terms of ITDs, in addition to the 0 ms ITD corresponding to the frontal sound position, we established a maximum ITD of 0.8 ms. This value was chosen to encompass the estimated temporal disparity of approximately 0.6–0.7 ms, which arises as a result of the head’s dimensions when sound is perceived directly from the lateral direction.

**Figure 1:**
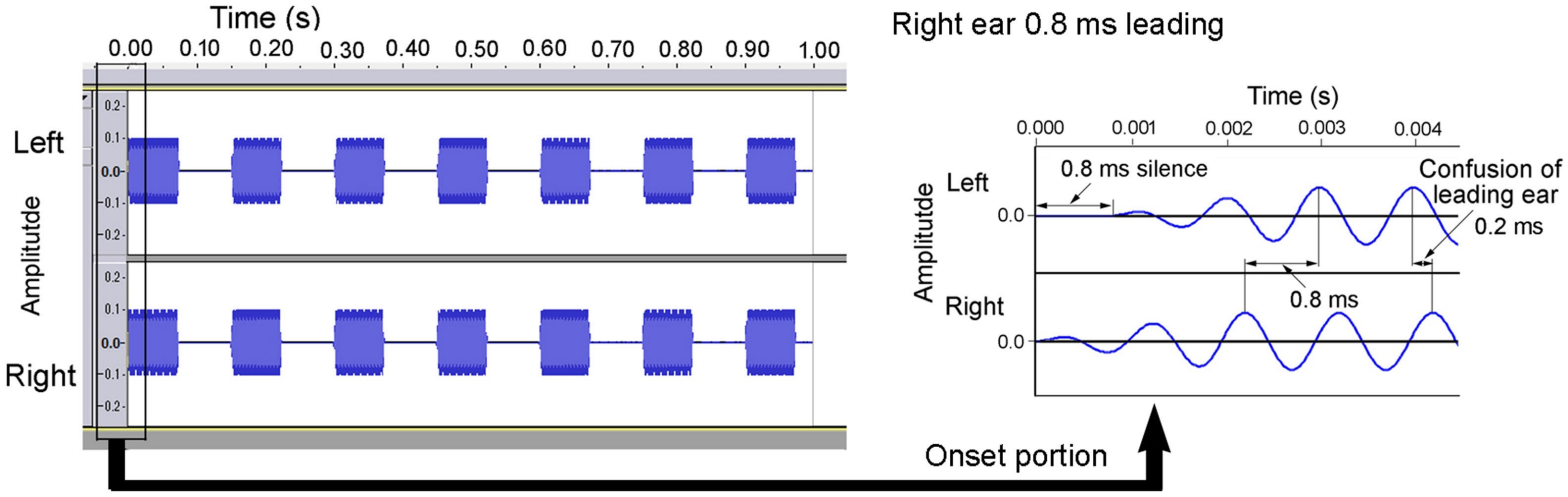
A sample of a 1 kHz sound wave leading in the right ear by 0.8 ms. When participants judged the direction based on the ongoing portion, they concluded that the sound was leading in the left ear by 0.2 ms because of phase reversion. Reproduced from Morita et al. (2023a) with the permission of the Acoustical Society of America.

Figure 1 illustrates an example of a sound wave leading to the right ear by 0.8 ms with a rise time of 2 ms (Morita et al., 2023a). At the onset of the sound, a silent period (0.8 ms) was exclusively presented to the left ear, and there was a shift in the waveform throughout the duration of the sound, thereby achieving the predetermined ITD. The amplitude of the sound at the onset increased gradually over 2 ms, yielding a rise time of 2 ms. At the end of the sound, the fall time was set to 2 ms. The sound was repeated seven times over the course of 1 s, characterized by an ON time of 74 ms and an OFF time of 76 ms. Both ears were presented with sounds at an equal sound pressure level of 60 dBA. At an ITD of 0.8 ms, a phase reversal occurred in the ongoing segment. In this context, the ear that experiences the leading sound at the onset is referred to as the “leading ear.”

**Figure 2:**
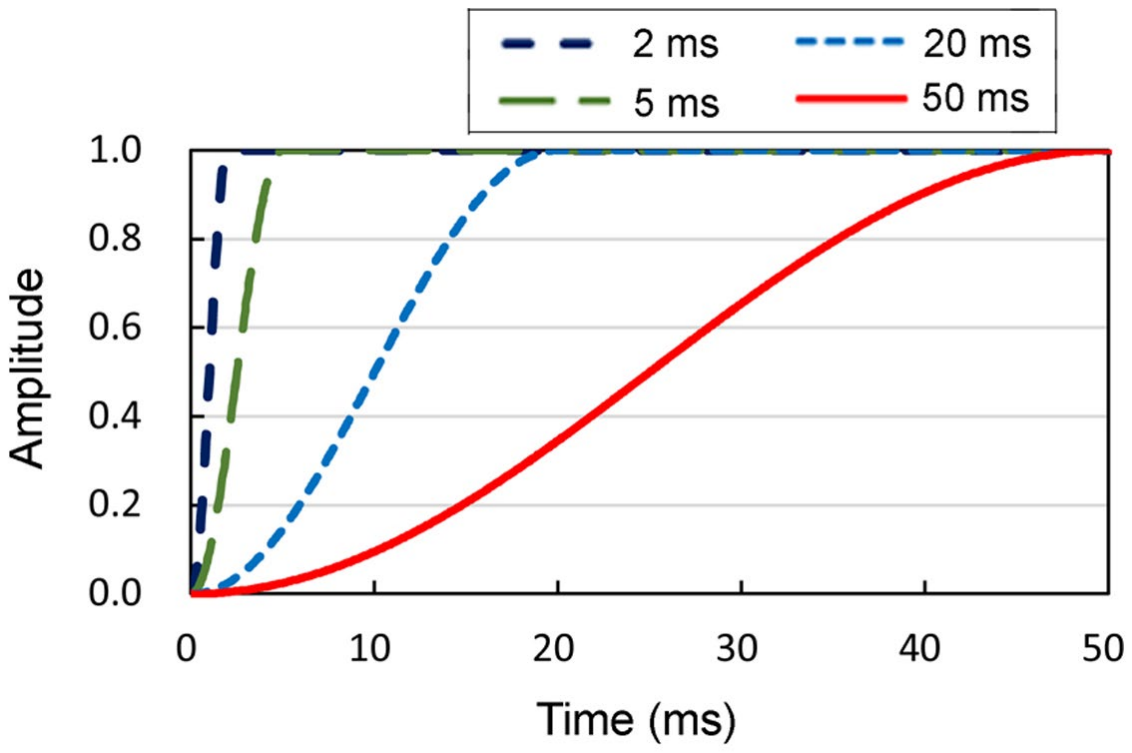
Four distinct shapes of smoothed onsets, each characterized by durations of 2, 5, 20, and 50 ms, respectively. The amplitude of the sound at the onset increased to the maximum, following each function. Conversely, the amplitude decay of the offset component of the sound was maintained for a constant duration of 2 ms.

The objective of the present study was to examine the effect of rise time on lateralization abilities. To accomplish this, we systematically manipulated the rise times of the onset portion by employing durations of 2, 5, 20, and 50 ms (Figure 2). Conversely, the amplitude decay of the offset component of the sound was maintained for a constant duration of 2 ms. The aforementioned ITD values and rise time values were determined referring to the literature (Kunov and Abel, 1981; Abel and Kunov, 1983).

**Figure 3:**
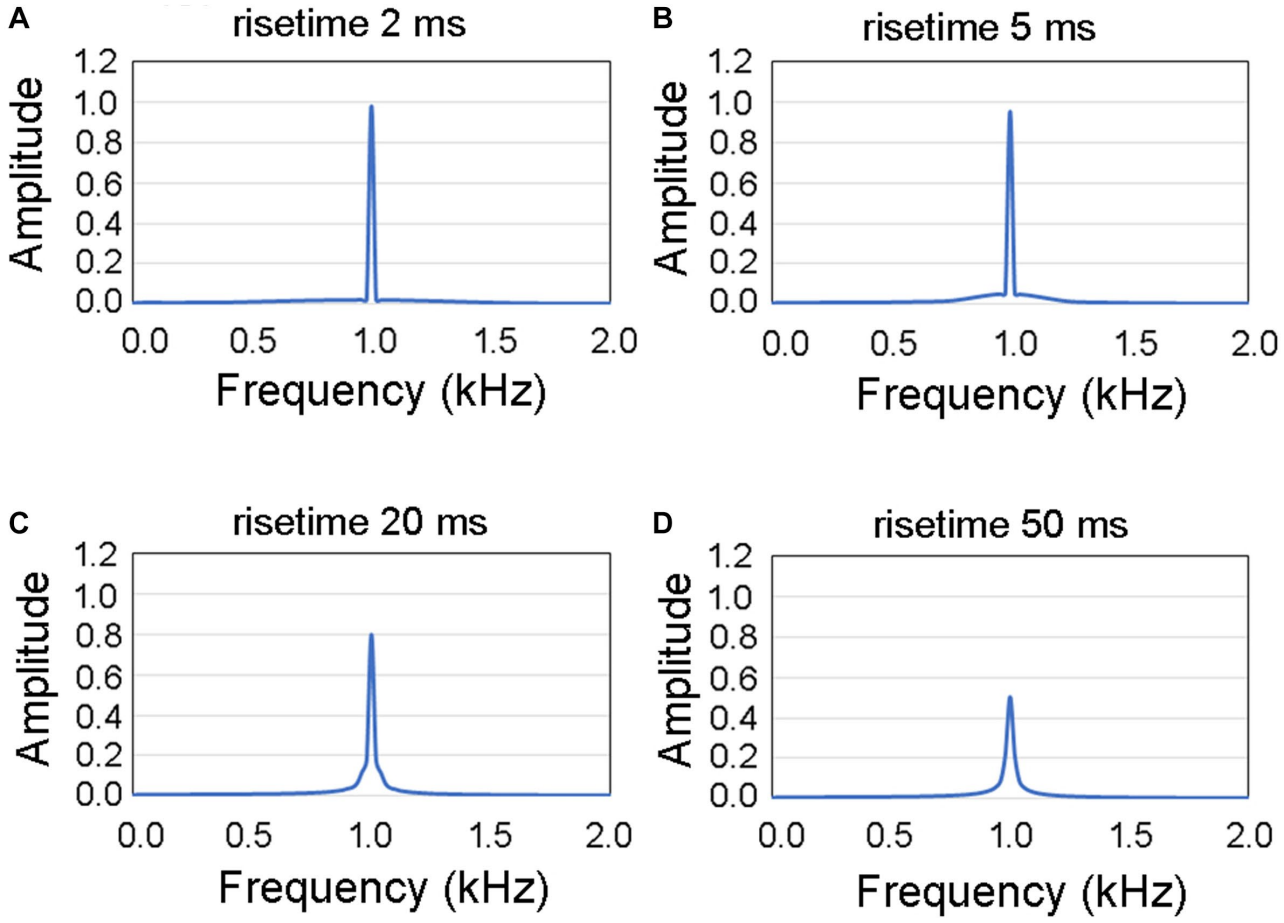
Spectral diagrams illustrating the characteristics of four distinct rise times within the initial 50 ms of the presented auditory stimulus. The spectral graphs represent rise times of **(A)** 2 ms, **(B)** 5 ms, **(C)** 20 ms, and **(D)** 50 ms. The extent of frequency dispersion remained relatively restricted to a frequency of 1 kHz.

Figure 3 illustrates the spectral characteristics of the signal waveforms observed within the initial 50 ms of sound. The spectrum corresponding to a rise time of 2 ms is depicted in the upper left panel (Figure 3A), whereas the subsequent panels represent the spectra for rise times of 5 ms (Figure 3B), 20 ms (Figure 3C), and 50 ms (Figure 3D). Upon visual examination, it became evident that with increasing rise time, there was a noticeable decrease in amplitude at a frequency of 1 kHz, whereas the extent of frequency dispersion remained relatively restricted. Consequently, we designated 1 kHz as the primary target frequency for the analysis.

### 2.3. Participants

Given that the primary objective of this study was to investigate the impact of four different rise time durations, the requisite number of participants was determined prior to conducting the experiment based on the following considerations. The expectation was that a repeated-measures analysis of variance (ANOVA) with within-group interactions would be employed, with two age groups as between-subject factors and four rise time durations as within-subject factors. The sample size was estimated using G*Power 3.1 (Faul et al., 2007; Mayr et al., 2007), with default assumptions employed for the calculations, including a medium effect size of 0.25, error probability (*α*) of 0.05, power value (1 − *β*) of 0.95, correlation of 0.5 among repeated measures, and a non-sphericity correction (*ε*) of 1. These values were used because of the lack of prior knowledge. The determined sample size was 36, equally divided between the two age groups, resulting in 18 participants per group. Consequently, the number of participants in each age group was set to 24 to provide a more flexible allowance for the sample.

The study sample consisted of 24 Japanese older adult participants (12 men and 12 women), with a mean age of 74.8 years (SD = 5.2 years, range = 67–91 years). Participants were recruited from the vicinity of the Chuo University in Tokyo, Japan, through the *Silver Jinzai Center* (a word-by-word translation would be Silver Human Resources Center; in an English translation made by the authors for convenience, Senior Citizens Job Bank) to ensure an equal number of men and women. The Senior Citizens Job Bank is a semi-public institution that has many older adults registered in its database; then, upon receiving requests from outside the institution, the Bank allocates older adults in various jobs, mainly in short-term jobs. We requested the Bank to assign older adults to participate in the experiments. The inclusion criteria were (1) being 65 years or older, (2) not using a hearing aid in their daily lives, (3) having no serious history of ear or brain disease, and (4) being healthy enough to travel to the university on their own. Additionally, 24 young participants (12 men and 12 women) with a mean age of 22.7 years (SD = 1.7 years, range = 19–26 years) were recruited from Chuo University. All were students (19 Japanese and 5 Chinese) at the Faculty of Science and Engineering. The criteria were the same as those for older adults, except that the required age for young participants was being around their 20s. Both young and older adults were recruited through convenience sampling.

At the time of the experiment all participants self-reported being in good health and denied any significant history of otological or neurological conditions.

### 2.4. Ethical considerations

Regarding ethical considerations, all study procedures were conducted while following the principles outlined in the Declaration of Helsinki. This study was approved by the Ethics Committee of the Chuo University (No. 2022-23). All participants provided written informed consent prior to participation.

### 2.5. Test procedure

In each trial, a test stimulus was presented 1 s after a reference stimulus of 1 kHz, with no ITD between the right and left ears and a rise time of 2 ms. Each trial consisted of a 60 dBA reference stimulus presented for 1 s, followed by a one-second blank period, and finally a 60 dBA test stimulus presented for 1 s. Participants were instructed to indicate the perceived direction of the test stimulus relative to the reference stimulus, choosing between the options “Left,” “Same,” or “Right.” Thus, the task employed a three-alternative forced choice procedure. No feedback was provided. The experiment was conducted intermittently from February to March 2022 for the older adult participants, and intermittently from September to October 2022 for the young participants.

We also investigated a potential factor that could influence the results of lateralization, namely the participants’ preferred ear (Porac and Coren, 1981; Strauss, 1986). Participants may have tended to respond in accordance with their preferred ears. The preferred ear was determined using a method where participants listened to quietly spoken words from a loudspeaker. In the upright position, the listener could not hear the speech; therefore, the person had to lean forward to hear it. The ear closest to the speaker at that time was identified as the preferred ear. Among the 24 older adult participants, 11 preferred their left ear and 13 their right ear. Among the 24 young participants, 8 preferred their left ear and 16 their right ear.

Additionally, the participants’ dominant hand, which indicates the hand they preferentially use, was determined based on their writing hand. All 24 older adult participants were right-handed, whereas among the young participants, 22 were right-handed and two were left-handed. While one theory suggests a relationship between left–right brain asymmetry and handedness (Satz et al., 1965; Porac and Coren, 1981; Hugdahl, 2004), it was impractical to conduct a meaningful analysis of handedness owing to the overwhelmingly large number of right-handed participants in our sample. Therefore, this study focused solely on analyzing the preferred ear.

### 2.6. Analysis method

At the beginning of the analysis, the normality of the data was assessed using a chi-square test, with the null hypothesis stating that the data followed a normal distribution. The analysis was conducted using JUSE-StatWorks, a statistical software developed by the Institute of JUSE in Japan.

To compare the two groups with no violation of normality assumptions, a two-tailed *t*-test was conducted, assuming a null hypothesis of equal means between the two groups. Conversely, nonparametric tests were employed in cases where normality assumptions were not met for a single group. Specifically, the Wilcoxon signed-rank sum test was used for paired samples, with the null hypothesis stating that the medians of the paired samples were equal. Additionally, the Mann–Whitney *U*-test was employed for independent samples, with the null hypothesis positing equal distributions between the two groups. When making comparisons for the four rise times found in sections 3.4 and 3.5, we used the nonparametric Friedman’s test. For correlational analysis (see section 3.7), Pearson’s correlation coefficient was calculated for the two groups with no violation of normality assumptions. In the case of violations, Spearman’s ρ was used. A two-tailed significance test was conducted assuming a null hypothesis of zero correlation between the groups. The statistical software IBM SPSS version 28 was used for all tests.

The level of statistical significance was set at 5% or 1% for all the analyses. If the observed difference was not statistically significant at the 5% level, it was considered non-rejectable and denoted as “not significant” (n.s.). Furthermore, to identify the important factors influencing the results, a neural network (NN) analysis was conducted using IBM SPSS version 28.

## 3. Results

### 3.1. Hearing ability of participants

**Figure 4:**
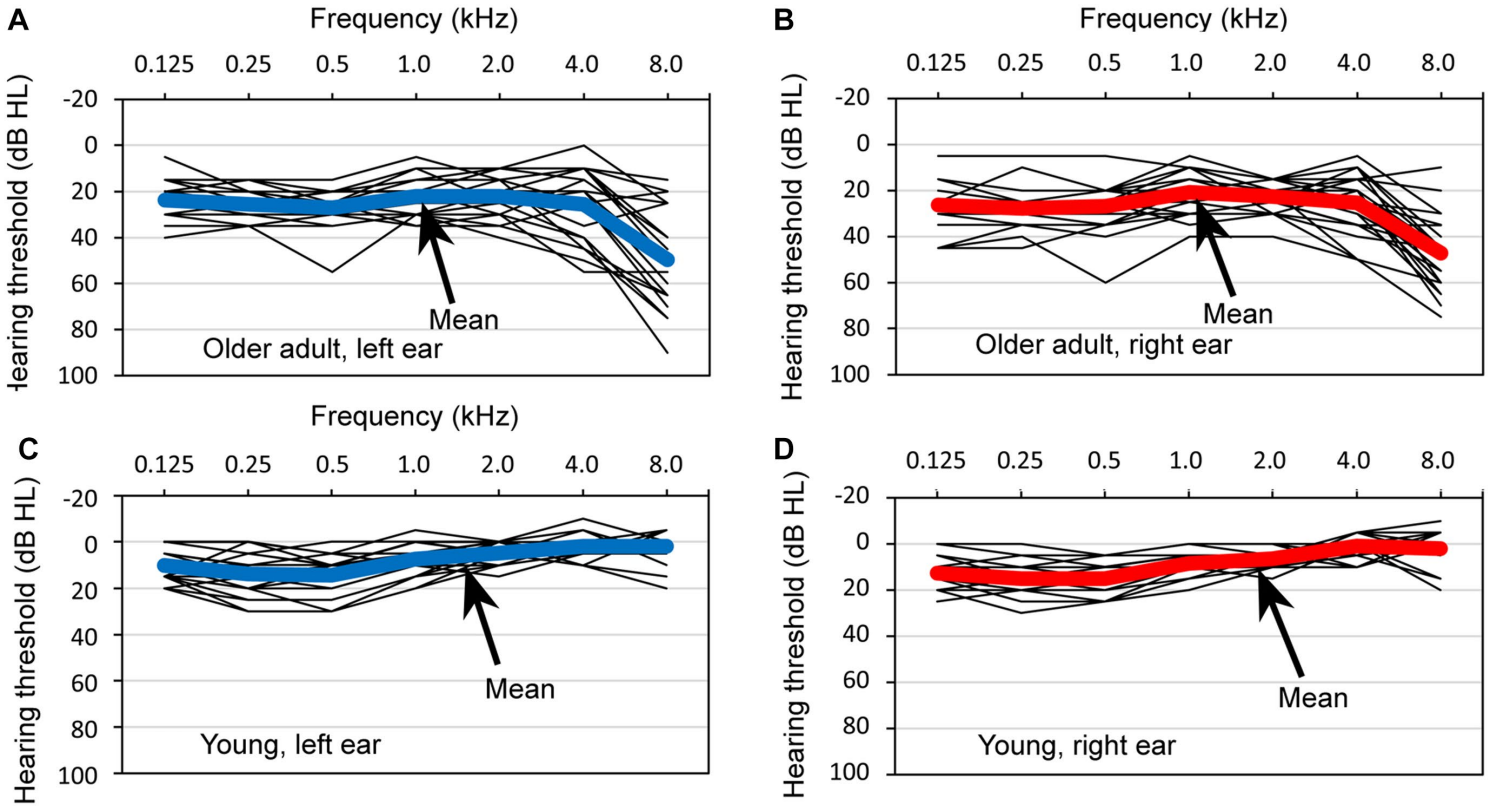
Hearing thresholds observed in a group of 24 older adult participants and 24 young participants. The thresholds are depicted for four distinct categories: **(A)** the left ear of the older adult participants, **(B)** the right ear of the older adult participants, **(C)** the left ear of the young participants, and **(D)** the right ear of the young participants. Within each graph, the thin lines represent the individual thresholds for each participant, with a step size of 5 dB hearing levels, and the bold line represents the mean threshold.

Figure 4 depicts the pure-tone thresholds of 24 older adult participants and 24 young participants measured using an audiometer (RION AA-M1C) across a frequency range of 0.125 to 8.0 kHz. In the figure, the thin lines represent the individual hearing thresholds, with a step size of 5 dB hearing levels (HLs), and the bold lines indicate the mean values. Specifically, Figures 4A–D display the thresholds for the left ears of older adult participants, right ears of older adult participants, left ears of young participants, and right ears of young participants, respectively.

When comparing HLs between the older adult and young participants, it was observed that HLs were poorer in older adults, particularly at higher frequencies. A visual examination of the data did not reveal any significant differences in the mean thresholds of the right and left ears of older adult participants, nor in the mean thresholds of the ears of young participants.

Regarding the hearing thresholds at a specific test frequency of 1 kHz, the following results were obtained: for the left ear of older adult participants, the mean threshold was 22.1 (SD = 8.2); for the right ear of older adult participants, the mean threshold was 20.8 (SD = 9.2); for the left ear of the young participants, the mean threshold was 7.5 (SD = 6.6); and for the right ear of the young participants, the mean threshold was 8.3 (SD = 5.5).

Given that the presented sound stimulus was a pure tone at 1 kHz, we conducted tests to examine significant differences in HLs specifically at this frequency. However, as the assumption of normality was violated in relation to HL at 1 kHz in the right ear of young participants, a nonparametric test was employed when analyzing groups that included HL in the right ear of young participants. The test results are as follows. First, there was a significant difference between young and older adult participants in the right ear (Mann–Whitney *U*-test, *p* < 0.001). Similarly, a significant difference between young and older adult participants was observed in the left ear [*t*(46) = 6.791, *p* < 0.001, Cohen’s d = 1.960]. Additionally, for young participants, the analysis did not provide sufficient evidence to reject the hypothesis that the medians of the hearing thresholds between the left and right ears were equal (Wilcoxon signed-rank sum test, *p* = 0.448). Similarly, for older adult participants, the analysis did not provide sufficient evidence to reject the hypothesis that the means of the hearing thresholds between the left and right ears were equal [*t*(23) = 1.030, *p* = 0.314, Cohen’s d = 0.210].

Consequently, it was determined that HLs at 1 kHz were poorer in older adult participants than in young participants. However, no significant difference was observed between the right and left ears in either older adult or young participants. Although some older adult participants exhibited poor hearing thresholds in the high-frequency range, they were not excluded from the experiment because of their ability to perform experimental tasks without difficulty.

### 3.2. Definition and distribution of correct answers

The participants were instructed to indicate the direction of the test sound relative to the reference sound using the descriptors Left, Same, or Right. In this context, “correct” judgment was operationally defined as the perception of lateralization toward the side of the leading ear at onset. For instance, when an ITD of 0.8 ms was employed, a phase reversal occurred between the onset and ongoing portions. Consequently, if the lateralization judgment was based solely on ongoing auditory information, the resulting judgment was considered “incorrect.” In other words, the response Right was classified as “correct” when the leading ear was positioned on the right side, and vice versa. At an ITD of 0.0 ms, the frequency of Same responses was utilized.

Upon evaluating the normality of the distribution of correct answers, it was found that normality could not be rejected in 21 of the 72 conditions, whereas it was rejected in 51 conditions (refer to Table 1 for older adult participants and Table 2 for young participants). Consequently, during the analysis of the results, a two-tailed *t*-test was performed when both compared groups exhibited a normal distribution, whereas a nonparametric test was employed when either one or both distributions deviated from normality. Accordingly, the initially planned ANOVA was replaced with a nonparametric test or *t*-test during the actual analysis process.

**Table 1:** Percentage of correct answers (%) for older adult participants observed under the conditions of ITD and rise time.

Age	ITD	Rise time	Mean (%)	Standard error (%)	Number of participants	Test of normality
Older adults	−0.8 ms	2 ms	55.0	8.2	24	n.s.
		5 ms	41.7	8.1	24	Rejected
		20 ms	15.8	5.4	24	Rejected
		50 ms	7.1	2.5	24	Rejected
	−0.6 ms	2 ms	86.7	5.3	24	Rejected
		5 ms	62.5	7.6	24	Rejected
		20 ms	33.8	7.1	24	n.s.
		50 ms	19.2	4.9	24	Rejected
	−0.4 ms	2 ms	91.7	4.6	24	Rejected
		5 ms	85.8	5.2	24	Rejected
		20 ms	67.9	6.8	24	n.s.
		50 ms	52.1	6.6	24	n.s.
	−0.2 ms	2 ms	76.3	5.1	24	Rejected
		5 ms	70.4	5.1	24	n.s.
		20 ms	68.8	5.9	24	Rejected
		50 ms	65.4	6.4	24	n.s.
	0 ms	2 ms	90.8	2.9	24	Rejected
		5 ms	92.1	2.9	24	Rejected
		20 ms	80.4	5.3	24	Rejected
		50 ms	75.8	5.8	24	Rejected
	0.2 ms	2 ms	86.3	3.9	24	Rejected
		5 ms	75.0	5.3	24	n.s.
		20 ms	71.7	5.9	24	Rejected
		50 ms	61.3	6.6	24	Rejected
	0.4 ms	2 ms	95.0	2.2	24	Rejected
		5 ms	94.6	3.0	24	Rejected
		20 ms	78.3	4.8	24	n.s.
		50 ms	64.6	6.5	24	Rejected
	0.6 ms	2 ms	93.8	2.7	24	Rejected
		5 ms	78.8	5.2	24	n.s.
		20 ms	50.4	6.8	24	n.s.
		50 ms	37.1	6.6	24	n.s.
	0.8 ms	2 ms	72.5	5.7	24	n.s.
		5 ms	55.8	7.6	24	Rejected
		20 ms	17.1	4.9	24	Rejected
		50 ms	13.8	4.9	24	Rejected

Negative ITD values indicate that the stimuli were presented first in the left ear, whereas positive ITD values indicate that the stimuli were presented first in the right ear. The outcomes of the normality test are indicated as “rejected” when the significance probability is equal to or less than *p* = 0.05, while “n.s.” is used for higher values, signifying that normality is not rejected. ITD, interaural time difference.

**Table 2:** Percentage of correct answers (%) for the young participants observed under the conditions of ITD and rise time (otherwise, the same as for Table 1).

Age	ITD	Rise time	Mean (%)	Standard error (%)	Number of participants	Test of normality
Young	−0.8 ms	2 ms	94.6	3.4	24	Rejected
		5 ms	85.8	4.7	24	Rejected
		20 ms	47.1	5.7	24	n.s.
		50 ms	24.6	4.2	24	Rejected
	−0.6 ms	2 ms	95.8	2.8	24	Rejected
		5 ms	92.1	3.3	24	Rejected
		20 ms	64.6	4.9	24	n.s.
		50 ms	39.6	6.0	24	n.s.
	−0.4 ms	2 ms	94.2	3.3	24	Rejected
		5 ms	88.8	3.7	24	Rejected
		20 ms	70.4	4.3	24	n.s.
		50 ms	53.3	5.2	24	n.s.
	−0.2 ms	2 ms	76.7	4.5	24	n.s.
		5 ms	70.0	5.2	24	Rejected
		20 ms	72.1	4.3	24	n.s.
		50 ms	59.6	5.3	24	Rejected
	0 ms	2 ms	95.4	2.6	24	Rejected
		5 ms	94.6	2.1	24	Rejected
		20 ms	77.1	4.7	24	n.s.
		50 ms	79.2	5.3	24	Rejected
	0.2 ms	2 ms	82.9	4.3	24	Rejected
		5 ms	76.3	4.2	24	Rejected
		20 ms	83.8	3.2	24	n.s.
		50 ms	83.8	4.1	24	Rejected
	0.4 ms	2 ms	96.7	2.2	24	Rejected
		5 ms	95.4	1.9	24	Rejected
		20 ms	94.6	2.2	24	Rejected
		50 ms	90.0	2.9	24	Rejected
	0.6 ms	2 ms	98.3	1.7	24	Rejected
		5 ms	95.4	2.6	24	Rejected
		20 ms	90.4	2.3	24	Rejected
		50 ms	82.5	4.5	24	Rejected
	0.8 ms	2 ms	96.3	3.3	24	Rejected
		5 ms	93.8	3.1	24	Rejected
		20 ms	79.2	4.2	24	Rejected
		50 ms	63.3	5.3	24	Rejected

ITD, interaural time difference.

### 3.3. Importance of each predictor in determining the NN

In the present experimental condition, the influence of seven factors on left–right responses was investigated. These factors included two types of age, four types of rise time, nine types of ITD, left ear hearing threshold at 1 kHz, right ear hearing threshold at 1 kHz, difference between the left ear and right ear hearing thresholds at 1 kHz, and preferred ears. Among these factors, age and preferred ear were treated as categorical variables, while the remaining factors were considered scale variables.

The impact of these explanatory variables on the observed lateralization outcomes was examined using an independent variable importance analysis within the NN framework. The experimental dataset consisted of 17,280 data derived from 48 participants, nine ITDs, four rise times, and 10 repetitions. The training set comprised 70% of the data, and the remaining 30% were reserved for the confirmation test. The output variables consisted of three units: Left, Same, and Right.

**Figure 5:**
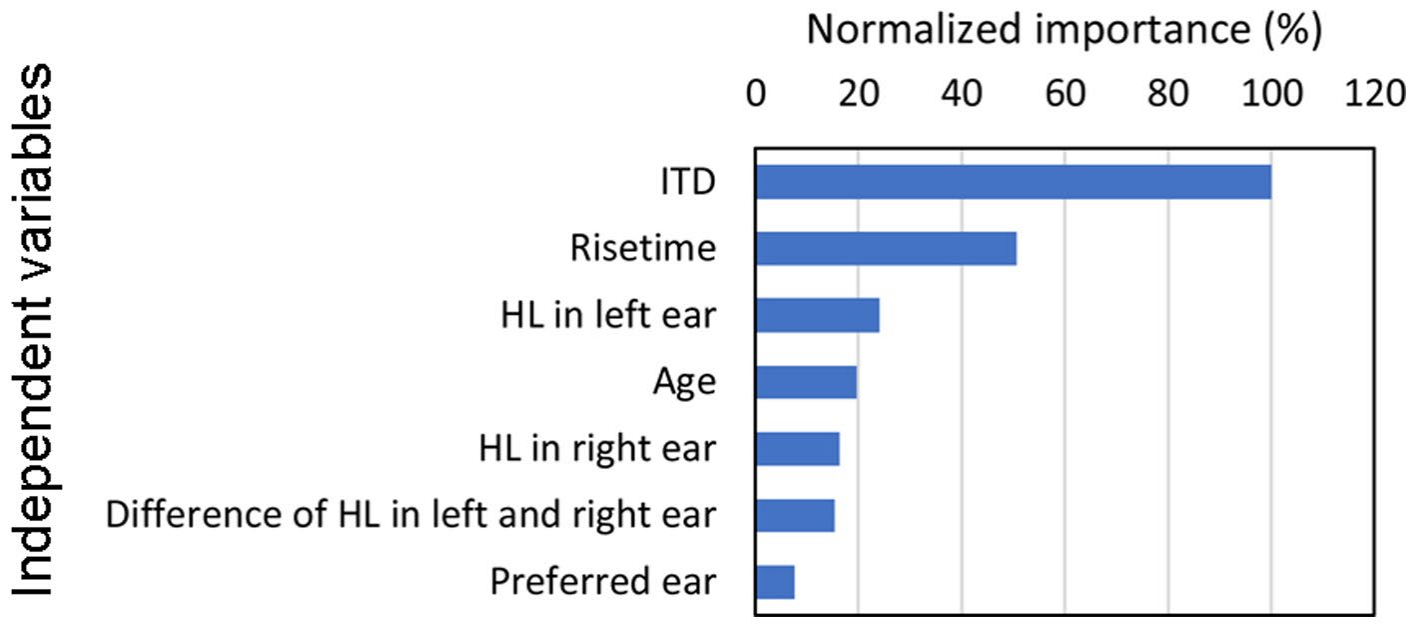
Relative significance of each predictor utilized during the construction of the NN, with normalization performed to establish the ITD value as 100. Building on our previous investigations of ITD (Morita et al., 2019, 2021, 2023b), this study focuses on exploring the impact of rise time, which represents, as shown in the figure, the second strongest association (i.e., normalized importance) with lateralization in our findings. HL, hearing level; ITD, interaural time difference; NN, neural network.

This analysis revealed the construction of an NN model with a single hidden layer containing six units. When this model was employed, the accuracy rates during the training and confirmation test phases were 73.3% and 73.5%, respectively, indicating sufficiently high estimation levels. Figure 5 presents the normalized importance of the independent variable for the seven explanatory variables, with each predictor’s value normalized in relation to the contribution of the ITD, which held the highest level of importance at 100.

The results indicated that the ITD exhibited the strongest association with lateralization. Conversely, hearing thresholds in the left and right ears as well as age displayed minimal relationships with lateralization. Moreover, the preferred ear demonstrated the least pronounced association with observed outcomes. Building on our previous investigations of ITD (Morita et al., 2019, 2021, 2023b), this study focuses on exploring the impact of rise time, which represents the second strongest association with lateralization in our findings.

### 3.4. Outline of correct answers of each ITD conditions

**Figure 6:**
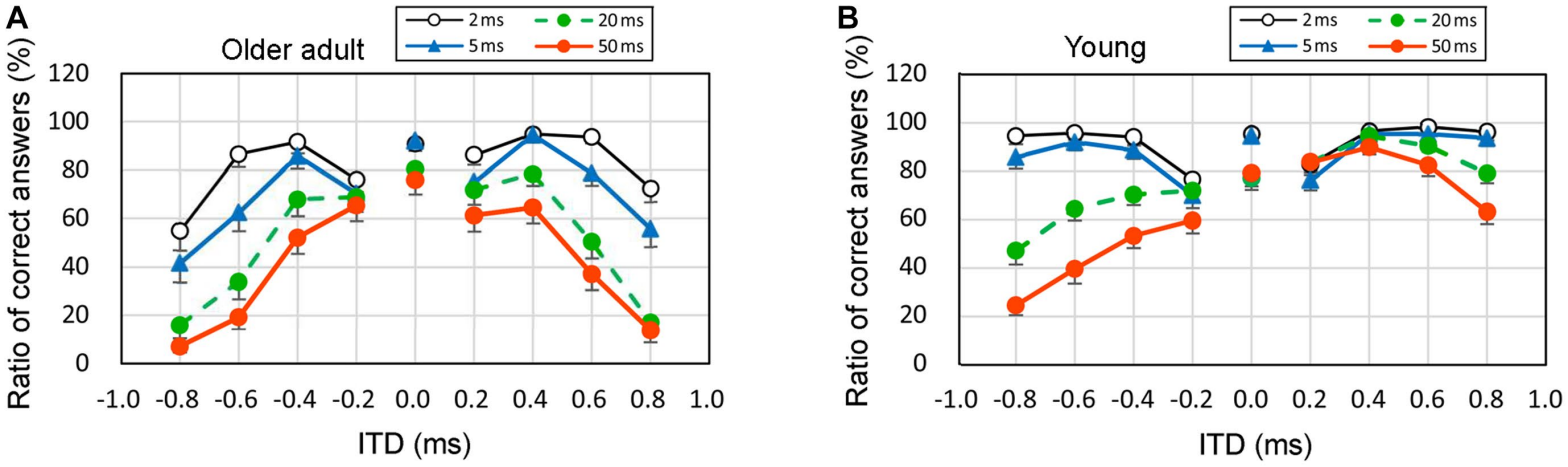
Percentage of correct answers when the sound of 1 kHz was presented with respect to four rise times, left panel **(A)**: older adult participants, right panel **(B)**: young participants. The horizontal axis displays ITD values, wherein negative values signify stimuli presented to the left ear first, while positive values indicate stimuli presented to the right ear first. Error bars mean the standard errors (SEs). ITD, interaural time difference.

To analyze the observed patterns, Figure 6 illustrates the average values of the percentage of correct answers among older adult participants (represented in the left graphs, denoted as Figure 6A) and young participants (represented in the right graphs, denoted as Figure 6B). The vertical axis represents the percentage of correct answers, whereas the horizontal axis represents the nine ITD conditions. Negative ITD values indicated that the stimuli were presented first in the left ear, whereas positive ITD values indicated that the stimuli were presented first in the right ear. The standard errors (SEs) are presented as error bars.

Upon visual examination, the overall trend indicated a lower correct answer rate for older adult participants than young participants. For example, the mean of the ratio of correct answers at an ITD of 0.8 ms and a rise time of 50 ms was 7.1% and 24.6% for older adult participants with left-ear precedence and young participants with left-ear precedence, respectively. Furthermore, as rise time increased, the correct answer rate decreased, even among young participants. This suggests that a longer rise time leads to inadequate recognition of the onset portion. Additionally, as the rise time increased, the left–right ear asymmetry became prominent among young participants. For instance, among young participants, the mean of the ratio of correct answers at an ITD of 0.8 ms and a rise time of 50 ms was 24.6% and 63.3% at left-ear precedence and right-ear precedence, respectively. Rigorous statistical analyses other than visual observation should be conducted based on the distribution of the ratio of correct answers, and these are described in the following paragraph and discussed in sections 3.5 and 3.6.

Figure 6 presents the mean values for simplicity. However, the normality assumptions concerning the distribution were violated for many conditions, as indicated in Tables 1, 2. Each age group comprised nine ITDs, and Friedman’s test was conducted for each ITD to examine potential variations in distribution based on rise time. The null hypothesis stated that the distributions of the four rise times were identical. Consequently, the null hypothesis could not be rejected for three specific conditions: 0.2 ms leading to the left ear in young participants (*p* = 0.125), 0.2 ms leading to the right ear in young participants (*p* = 0.068), and 0.2 ms leading to the left ear in older adult participants (*p* = 0.262). However, for the remaining ITDs, the null hypothesis based on rise time was rejected. In summary, a significant difference in the ratio of correct answers exists across various conditions owing to the rise time.

The Friedman’s test results described in the above paragraph indicate that when the ITD was short, the difference by rise time was small, but as the ITD increased to 0.6 or 0.8 ms, the effect of rise time became apparent. Since the purpose of this study was to investigate whether the length of rise time affects the perception of the onset portion, in the subsequent sections, we discuss the results for the ratio of correct answers at an ITD of 0.8 ms as an example.

### 3.5. Ratio of correct answers at an ITD of 0.8 ms and comparing four rise times

**Figure 7:**
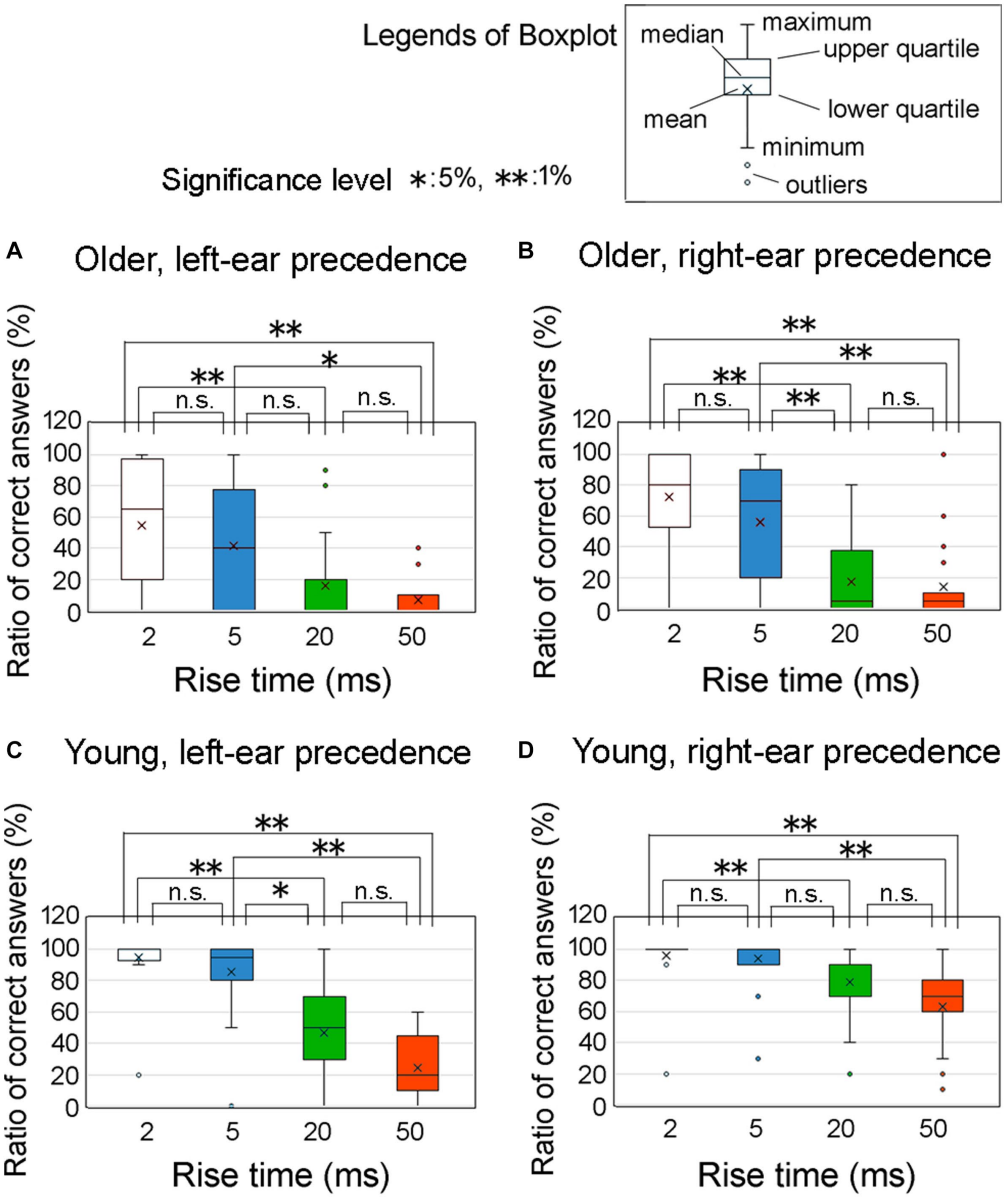
Percentage of correct answers at an ITD of 0.8 ms for the four rise time conditions. In the figure, **(A)** refers to older adult participants with left-ear precedence, **(B)** refers to older adult participants with right-ear precedence, **(C)** refers to young participants with left-ear precedence, and **(D)** refers to young participants with right-ear precedence. These conditions were examined at an ITD of 0.8 ms. Asterisks denote significant differences observed among the rise time conditions, as determined by Friedman’s test. To address the issue of multiple comparisons, the Bonferroni correction was employed. The legends for the boxplot are shown in the figure. ITD, interaural time difference.

Figure 7 illustrates the ratio of correct answers at an ITD of 0.8 ms among older adult participants with left-ear precedence (upper left panel, labeled as Figure 7A), older adult participants with right-ear precedence (upper right panel, labeled as Figure 7B), young participants with left-ear precedence (lower left panel, labeled as Figure 7C), and young participants with right-ear precedence (lower right panel, labeled as Figure 7D). The horizontal axis represents the four distinct rise times. Asterisks indicate a significant distinction between the rise-time conditions, as determined by Friedman’s test under the null hypothesis that the distributions of the groups are equivalent. Bonferroni correction was employed to account for multiple comparisons. The null hypothesis was rejected for many combinations, signifying a significant relationship between rise time and the ratio of correct answers.

This graph provides clear evidence that older adult participants exhibited greater variability in the ratio of correct answers than young participants. For young individuals, the graph shows that the proportion of correct responses was higher in the case of right-ear precedence (Figure 7D) than left-ear precedence (Figure 7C) when the rise times were 20 and 50 ms. To compare the differences in variability of the ratio of correct answers for Figure 7 (i.e., Figures 7A–D), the data were aggregated for each group. However, since the ratio of correct answers did not follow a normal distribution, we compared them using a nonparametric method. One way was to subtract the value of 25 percentiles (lower-quartile) from the value of 75 percentiles (upper-quartile), which means the vertical axis of the box. This procedure yielded the following results: 60% for older adult participants with left-ear precedence; 80% for older adult participants with right-ear precedence; 70% for younger participants with left-ear precedence; and 30% for young participants with right-ear precedence. These findings show that the variability in young participants with right-ear precedence was obviously smaller than that for the other groups. Further examination of the disparities between the leading ears is addressed in the subsequent section.

### 3.6. Ratio of correct answers at an ITD of 0.8 ms and comparing the age groups and leading ear

**Figure 8:**
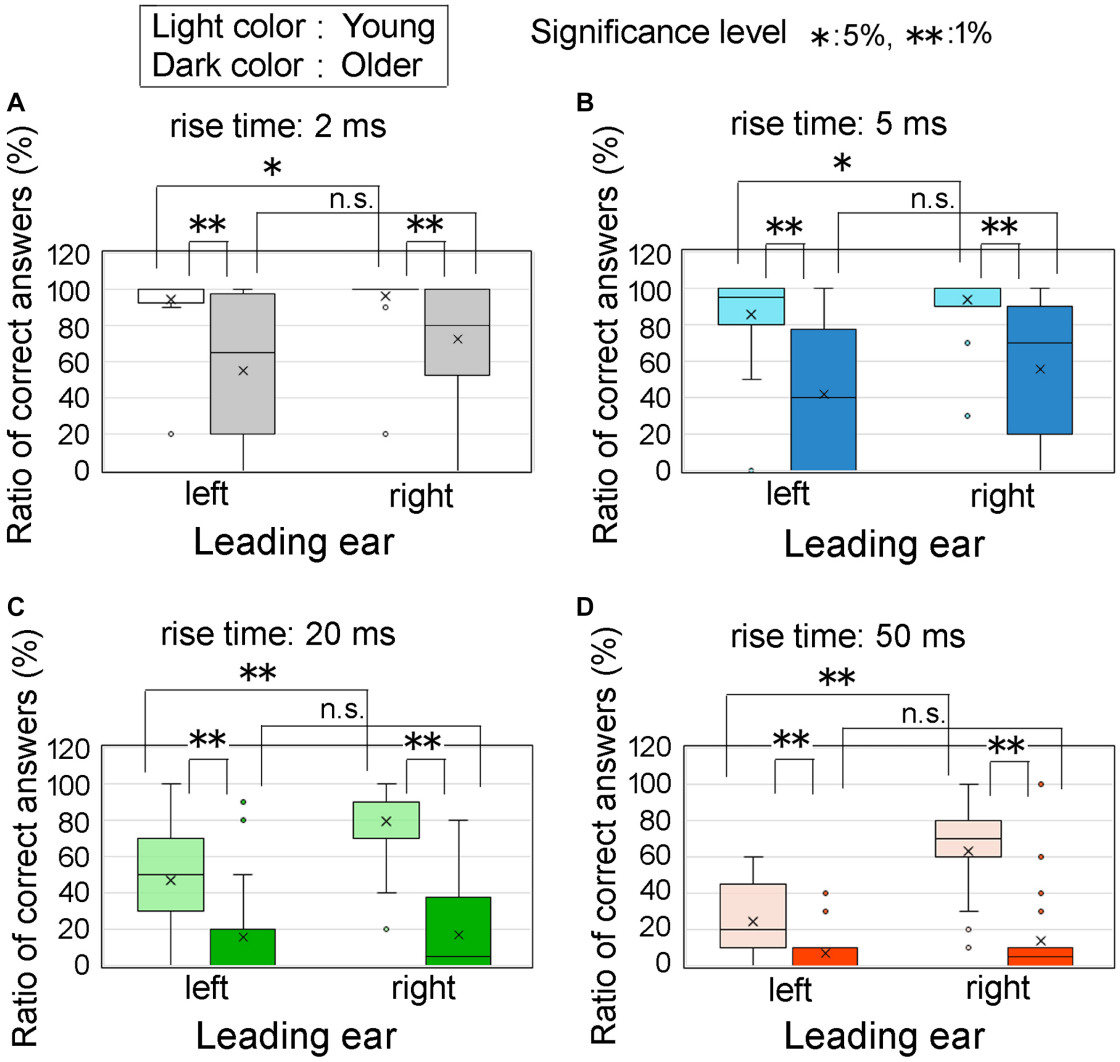
Percentage of correct answers at an ITD of 0.8 ms for the age groups and leading ear. In the figure, Panels **(A–D)** represent the different leading ears and the rise times of 2, 5, 20, and 50 ms, respectively. The horizontal axis represents the leading ears, specifically the left and right ears. The light and dark colored boxes represent the results obtained from young participants and older adult participants, respectively. Asterisks denote statistically significant differences in test conditions, which were determined using *t*-tests or nonparametric tests such as the Wilcoxon signed-rank sum test for paired samples or the Mann–Whitney test for two-independent samples. The legends for the boxplot are the same as those shown in Figure 7. ITD, interaural time difference.

To provide a comparative analysis of the effects of age group and leading ear, we investigated the ratio of correct answers using an ITD of 0.8 ms. Figure 8 displays the ratios of correct answers corresponding to four different rise times: 2 ms (upper left, Figure 8A), 5 ms (upper right, Figure 8B), 20 ms (lower left, Figure 8C), and 50 ms (lower right, Figure 8D). The light colored and dark colored boxes represent young and older adult participants, respectively. The horizontal axis represents the leading ear.

Regarding the statistical analysis, when examining the older adult group at a rise time of 2 ms, the assumption of normality was not rejected for the ratio of correct answers in both the right ear-leading and left ear-leading conditions. Consequently, a *t*-test was used to assess the combinations of these conditions. However, for the other conditions, the assumption of normality was rejected for the ratio of correct answers, necessitating the use of nonparametric tests. Specifically, we used the Wilcoxon signed-rank sum test for paired samples and the Mann–Whitney *U-*test for two independent samples.

Compared with the young participants, the older adult group exhibited a decrease in the ratio of correct answers across all rise times. In the case of young participants, the ratio of correct answers was significantly higher when the right ear preceded the left ear, as evidenced by the conspicuous trend observed in Figure 8D. In other words, the REA was clearly demonstrated. However, for older adult participants, no distinction was observed in the ratio of correct answers based on the leading ear, implying that the REA phenomenon did not hold true for this age group.

### 3.7. Influence of hearing thresholds on lateralization judgment

The aforementioned disparity in the ratio of correct answers may be associated with variations in hearing thresholds, raising the question of whether the participants’ hearing thresholds are related to their behavioral performance in the corresponding ear-leading condition. For example, there might be a significant correlation between the participant’s left-ear hearing threshold and their ratio of correct answers in the left ear-leading condition, and vice versa.

**Figure 9:**
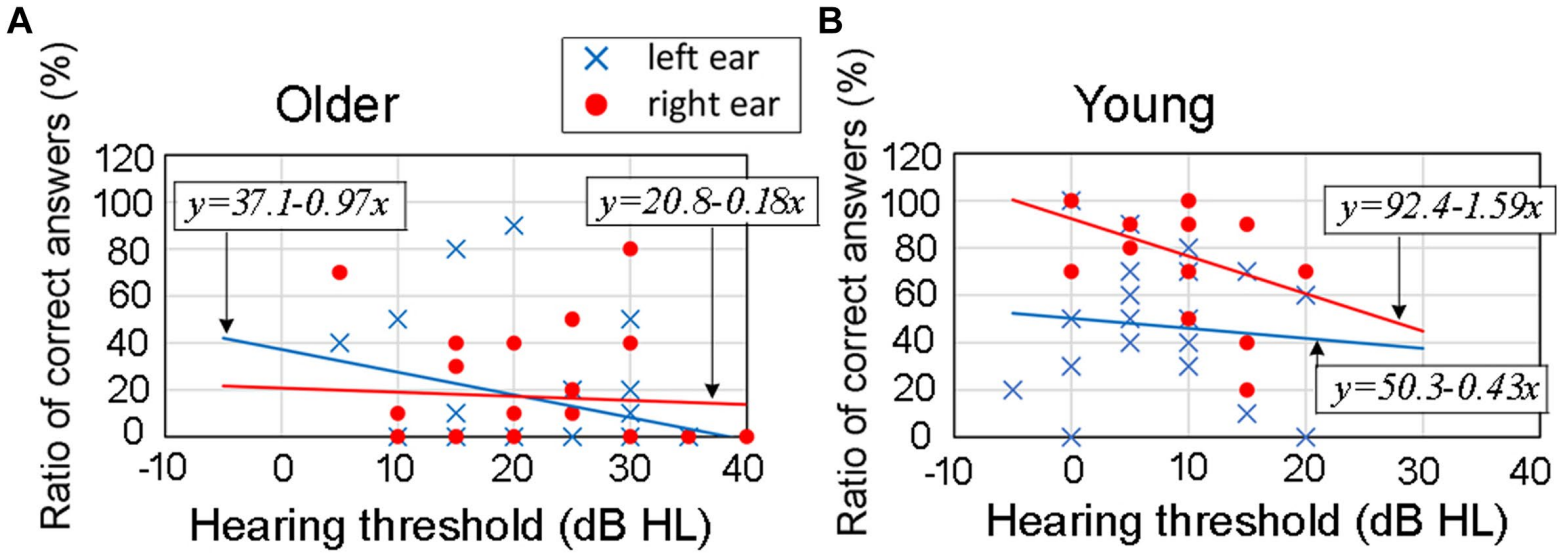
Results for the correlation between hearing thresholds and the ratio of correct answers at an ITD of 0.8 ms and rise time of 20 ms. In the figure, panel **(A)** refers to older adult participants, panel **(B)** refers to young participants. Blue marks indicate data for the left ear and red marks for the right ear. The figures include the regression equations for both the left ear (blue line) and right ear (red line) for each age group. The results of correlational analysis are shown in Table 3. ITD, interaural time difference.

To illustrate this relationship, Figure 9 indicates samples of the correlation between hearing thresholds (horizontal axis) and the ratio of correct answers (vertical axis) at an ITD of 0.8 ms and rise time of 20 ms. Figure 9A shows the correlation for older adult participants, while Figure 9B shows the same relationship for young participants. Blue marks indicate data for the left ear and red for the right ear. Figure 9 includes the regression equations for both the left ear (blue line) and right ear (red line).

It was presumed that as the hearing thresholds worsened (moved toward the right direction on the horizontal axis), the ratio of correct answers would decrease. However, Figure 9 suggests that there might not be a strong relationship between hearing thresholds and the ratio of correct answers in the corresponding ear-leading condition. Therefore, we conducted a correlational analysis to examine this relationship.

Table 3 indicates the correlation results for 64 conditions comprising the two age groups, four ITDs for each leading ear, and four rise-time conditions. Among them, the null hypothesis was rejected in three conditions: 0.8 ms leading to the left ear at a rise time of 2 ms in the older adult participants; 0.6 ms leading to the left ear at a rise time of 2 ms in young participants; and 0.8 ms leading to the right ear at a rise time of 20 ms in the young participants. However, in the remaining 61 conditions, the null hypothesis was not rejected. Therefore, it was concluded that there was no significant correlation between the hearing thresholds of the participants and the ratio of correct answers in the corresponding ear-leading condition.

**Table 3:** Correlation coefficients for the associations between the participants’ hearing thresholds and the ratio of correct answers in the corresponding ear-leading condition.

Age	Left/right ear	ITD	Rise time			
			2 ms	5 ms	20 ms	50 ms
Older adults	Left	−0.8 ms	−0.411^*^	−0.271	−0.280	−0.270
		−0.6 ms	−0.400	−0.374	−0.381	0.052
		−0.4 ms	−0.095	−0.230	−0.126	−0.100
		−0.2 ms	−0.055	0.006	0.161	0.022
	Right	0.2 ms	0.147	−0.092	0.235	0.123
		0.4 ms	−0.285	−0.072	0.037	0.240
		0.6 ms	−0.016	−0.221	−0.030	0.221
		0.8 ms	−0.366	−0.098	−0.090	−0.017
Young	Left	−0.8 ms	−0.207	−0.013	−0.100	0.214
		−0.6 ms	−0.436^*^	−0.248	−0.213	−0.175
		−0.4 ms	−0.176	−0.335	0.086	0.104
		−0.2 ms	−0.193	0.005	0.023	0.288
	Right	0.2 ms	0.161	−0.128	0.099	−0.092
		0.4 ms	0.235	0.175	−0.150	0.027
		0.6 ms	−0.065	0.264	0.112	−0.079
		0.8 ms	−0.262	0.274	−0.417^*^	−0.119

Pearson’s correlation coefficient was calculated for the two groups with no violation of normality assumptions. In the case of violations, Spearman’s ρ was used. A two-tailed significance test was conducted assuming a null hypothesis of zero correlation between the hearing thresholds and the ratio of correct answers with a significance level set at 5%. Gray cells with asterisk indicate significant differences. ITD, interaural time difference.

From a different perspective, we examined the possibility that the difference between left and right hearing thresholds affects the ratio of correct answers. We compared two groups, namely one comprising participants with a hearing threshold of 5 dB or better in the left ear at 1 kHz, and the other consisting of participants with superior hearing in the right ear. In instances where sounds originated from the left ear, participants were expected to respond with Left if they detected the onset portion using their left ear. It was hypothesized that individuals with superior hearing thresholds in the left ear, as compared to the right ear, would exhibit enhanced recognition of the onset portion. Consequently, they would be more inclined to answer with Left. In contrast, the opposite effect would be observed when the sounds initially appeared in the right ear.

Among older adult participants, the distribution based on their hearing thresholds was as follows: six individuals had better hearing in the left ear than in the right ear, 10 individuals had superior hearing in the right ear, and eight individuals exhibited equal hearing thresholds in both ears. For younger participants, these numbers were 11, eight, and five, respectively.

**Figure 10:**
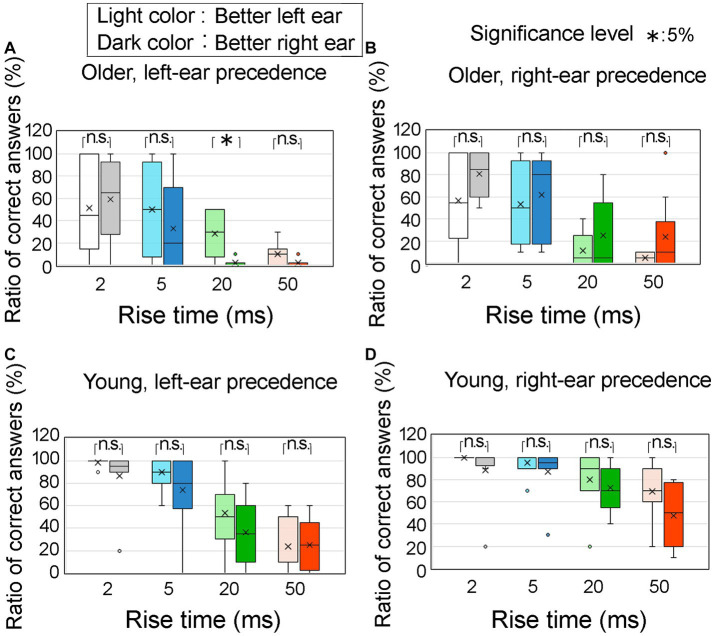
Percentage of correct answers at an ITD of 0.8 ms, focusing on the superior auditory abilities of participants. The analysis distinguishes between different conditions: **(A)** older adult participants with left-ear precedence, **(B)** older adult participants with right-ear precedence, **(C)** young participants with left-ear precedence, and **(D)** young participants with right-ear precedence. The utilization of light colored and dark colored boxes in the figure represent the better left and right ears, respectively. The horizontal axis corresponds to four distinct rise times. Notably, if we exclude a particular case (i.e., the rise time of 20 ms for older adult participants with left-ear precedence; **A**), the Mann–Whitney test indicates no significant differences in the remaining comparisons between the better left and right ears. The legends for the boxplot are the same as those in Figure 7. ITD, interaural time difference.

Figure 10 illustrates the ratio of correct answers at an ITD of 0.8 ms, specifically focusing on the superior ear of the participants under four conditions: left ear-leading for older adult participants (Figure 10A), right ear-leading for older adult participants (Figure 10B), left ear leading for young participants (Figure 10C), and right ear leading for young participants (Figure 10D). The left and right ears are represented by light colored and dark colored boxes, respectively. The horizontal axes represent the four rise times. With the exception of one case (at a rise time of 20 ms in the left ear-leading condition for older adults; Figure 10A), no significant differences were observed between the superior left and right ears comparisons, as determined by Mann–Whitney *U-*test.

Contrary to our initial expectations, our findings did not support the hypothesis that individuals with better hearing in the left ear would exhibit a higher ratio of correct answers in the left ear-leading condition, and vice versa.

### 3.8. Influence of preferred ear on lateralization judgment

Lateralization performance may be influenced by the participant’s preferred ear according to prior evidence (Porac and Coren, 1981; Strauss, 1986), suggesting that superior lateralization performance may occur when the right ear is the preferred ear and the test sound is initially presented to the right ear, and vice versa. As previously stated, 11 and 13 of the older adult participants preferred their left and right ear, respectively, while 8 and 16 of the young participants preferred their left and right ear, respectively.

**Figure 11:**
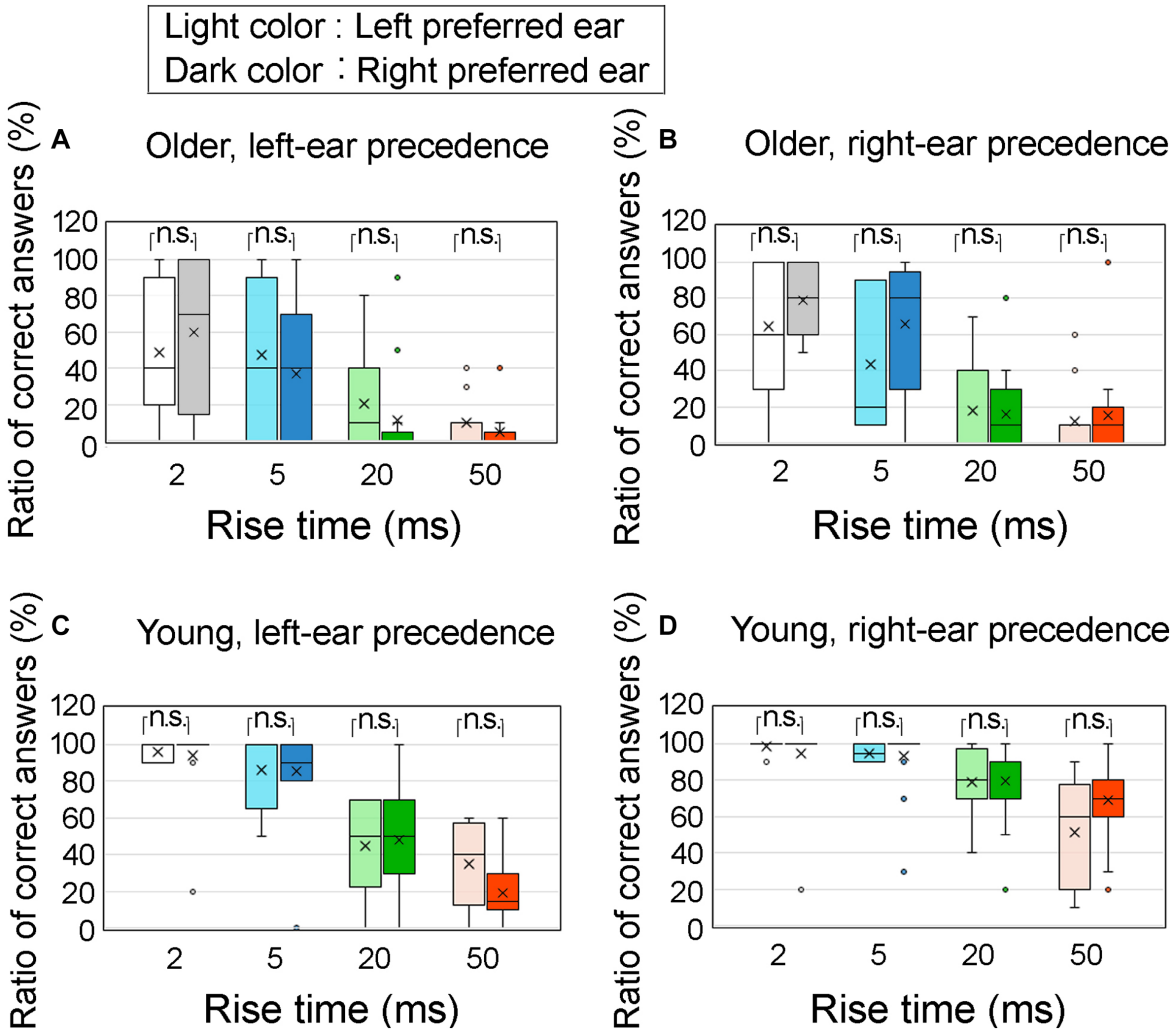
Percentage of correct answers at an ITD of 0.8 ms, specifically pertaining to the preferred ear of the participants. The older adult participants were divided into two groups: **(A)** older adult participants with left-ear precedence, **(B)** older adult participants with right-ear precedence, **(C)** young participants with left-ear precedence, and **(D)** young participants with right-ear precedence. The preferred left ear and preferred right ear are denoted by the light and dark colored boxes, respectively. The horizontal axis represents four different rise times. Statistical analysis using the Mann–Whitney test revealed no significant differences between the preferred left and right ear conditions. The legends for the boxplot are the same as those in Figure 7. ITD, interaural time difference.

Figure 11 displays the correct answer ratios at an ITD of 0.8 ms, considering the preferred ears of the participants. Figure 11A shows results for older adult participants with left-ear precedence; Figure 11B for older adult participants with right-ear precedence; Figure 11C for young participants with left-ear precedence; Figure 11D for young participants with right-ear precedence. The preferred left and right ears are represented by light colored and dark colored boxes, respectively. The horizontal axis represents the four rise times.

To examine the influence of the preferred ear, a Mann–Whitney U test was conducted to compare the correct answer ratios between the left and right preferred ears. The results indicated no significant differences across all conditions. Therefore, it can be inferred that the impact of preferred ear on correct answer ratio was minimal.

## 4. Discussion

### 4.1. Influence of hearing ability and preferred ear on lateralization performance

Regarding hearing thresholds as a factor influencing lateralization performance, it is widely accepted that differences in hearing thresholds have minimal impact on sound localization performance (Jerger and Jordan, 1992; Otte et al., 2013; King et al., 2014; Morita et al., 2023a). Consistent with previous research, the results of the present study indicate that there was no significant correlation between the hearing thresholds of the participants and the ratio of correct answers in the corresponding ear-leading condition (Table 3). Furthermore, the disparity in hearing thresholds between the left and right ears was associated with lateralization performance in older adult participants in a single experimental condition, whereas the influence of this disparity on lateralization was predominantly insignificant across most conditions (Figure 10).

In relation to the association between hearing thresholds and REA observed in young participants, no noticeable disparity was observed in the mean hearing thresholds between the left and right ears, and the auditory capabilities in the right ear did not surpass those in the left ear. Consequently, the difference in hearing thresholds between the ears failed to account for the observed REA in young participants.

The preferred ear is another factor that must be considered. Porac and Coren (1981) investigated the relationship between dichotic listening test performance and ear preference and found that the association between dichotic listening and ear preference was not robust. In line with this finding, the results of the present study suggest a minor contribution of the preferred ear, as indicated by the importance analysis conducted using the NN analysis. Moreover, as depicted in Figure 11, the preference for the left or right ear exhibited no significant association with the ratio of correct answers during lateralization. Consequently, the impact of the preferred ear on lateralization performance, particularly the REA observed in young participants, was considered negligible.

### 4.2. Age-related changes in the REA

Since Kimura’s (1967) initial investigations of the dichotic listening test, numerous publications have emerged in the field of language-related REA. These studies have addressed various aspects of language REAs and made significant contributions to the understanding of this phenomenon (Hugdahl, 2011). Additionally, investigations have indicated that REAs can be elicited in response to rapid frequency transitions, owing to the reduced response of the right auditory cortex (Belin et al., 1998). In relation to the impact of aging on REAs, several studies have reported a sustained or even intensified REA effect (Gelfand et al., 1980; Jerger and Jordan, 1992; Tadros et al., 2005; Takio et al., 2009; Westerhausen and Kompus, 2018). Conversely, some studies have presented evidence suggesting the absence of the REA in aging populations (Hugdahl, 2004). These pieces of evidence showcase that the effects of aging on REAs remain unclear.

There is an ongoing debate regarding the factors associated with REAs, particularly the influence of peripheral auditory function and central brain processing (Divenyi and Haupt, 1997; Gootjes et al., 2004; Tadros et al., 2005; Westerhausen et al., 2015). Based on the findings reported by Tadros et al. (2005), in the presbycusis group, the amplitudes of otoacoustic emissions, which serve as indicators of peripheral function, were higher in the left ear than in the right ear. In contrast, in the realm of speech perception, which is closely associated with central function, the REA was evident. Drawing on research pertaining to attention allocation toward specific ears, Gootjes et al. (2004) hypothesized that aging may lead to a decline in corpus callosum functioning, impacting both the cerebral hemispheres and the interhemispheric connections. In a similar vein, Westerhausen et al. (2015) postulated that the decline in performance for stimuli presented to the left ear, rather than improved performance for stimuli presented to the right ear, was associated with deterioration of the corpus callosum.

An alternative viewpoint pertaining to hemispheric asymmetry of the brain posits that distinct regions within the brain are dedicated to language processing. Notably, Broca’s area is predominantly associated with speech production, whereas Wernicke’s area is primarily responsible for speech comprehension, both of which are in the left hemisphere (Peelle and Wingfield, 2022). For instance, Hugdahl (2004) conducted a dichotic listening test on a sample of 16 individuals with lesions in the left frontal lobe and 10 individuals with lesions in the right frontal lobe. This study revealed that patients with left-sided lesions failed to exhibit the anticipated REA, indicating an asymmetry in language processing. Furthermore, language-related functions tended to decline with age. Rotte (2005) used functional magnetic resonance imaging to investigate brain activity during the visual presentation of language. The findings indicate that activity within the Broca’s and Wernicke’s areas, associated with language processing, diminish with advancing age.

A noteworthy outcome of our current investigation on pure-tone lateralization was the confirmation of the REA not only in language processing, but also in pure-tone lateralization among young participants. We observed less variation in the responses for the right ear among young participants, suggesting that the right ear has better performance than the left ear. Moreover, our results demonstrated a decline in the REA with increasing age. These findings align with those of Divenyi and Haupt (1997) and Tadros et al. (2005), who also suggested that peripheral function plays a role in pure-tone lateralization. However, our previous research implied that the REA in pure tones is not exclusively tied to peripheral function.

Morita et al. (2023a) conducted two kinds of auditory tests, namely lateralization test and event-related potential measuring test, with 19 older adult and 14 young participants. In the lateralization test, participants were presented with sound stimuli with an ITD in the right or left ear, and required to indicate the sound direction. The test conditions were the same as those for the experiment presented in the current study, except that the ITD was limited to 0.4 and 0.8 ms, rise time was limited to 2 ms, and the response options were Left or Right (i.e., a two-way choice). Regarding an ITD of 0.8 ms, in which a phase reversal between the onset and ongoing portions was introduced, seven older adult participants responded “Right” to a sound leading in the left ear (i.e., incorrect response) and 12 older adult participants responded “Left” (i.e., correct response). Meanwhile, to a sound leading in the right ear, seven and 12 participants responded “Left” (i.e., incorrect response) and “Right” (i.e., correct response), respectively. Among the seven participants who answered the opposite direction when the left ear was leading and the seven who answered the opposite direction when the right ear was leading, three participants overlapped.

In the event-related potential measuring test, we conducted an oddball task to investigate the neural responses of participants to auditory stimuli of a 1-kHz pure tone with ITDs. Participants viewed a silent animation video on a 19-inch computer screen at a viewing distance of approximately 85 cm. The standard stimuli comprised sounds without any ITD between the right and left ears, while the deviant stimuli were presented with two ITD conditions: 0.4 and 0.8 ms leading either to the right or left ear. To examine automatic brain responses, the deviant stimuli with ITD were randomly interspersed among the frequent standard stimuli. The stimuli (i.e., 90% standard and 10% deviant stimuli) were presented at a rate of one tone per 500 ms. Each participant was exposed to 900 standard and 100 deviant stimuli for each ITD condition. Electroencephalograms were recorded using a BrainAmp DC (Brain Products GmbH, Germany) at a sampling rate of 1 kHz and 16-bit resolution from Ag/AgCl electrodes (F3, Fz, F4, C3, Cz, C4, P3, Pz, and P4) following the international 10/20 system. The analysis focused on mismatch negativity (MMN), an automatic neural response to auditory stimuli that deviate from preceding stimuli (Näätänen, 2000; Näätänen et al., 2012; Luck, 2014). To delineate MMN, standard-stimulus event-related potentials were subtracted from the corresponding deviant-stimulus event-related potentials. The MMN values were obtained by taking the average of 100–200 ms for the MMN waveforms at C3, Cz, and C4 in the central area, and F3, Fz, and F4 in the frontal area. The event-related potentials in the parietal area, which are considered less related to auditory MMN, were not analyzed. Regarding the event-related potentials data for all conditions, normality was not rejected nor was equal variance. The original study provides more details on the methodology used (Morita et al., 2023a).

**Figure 12:**
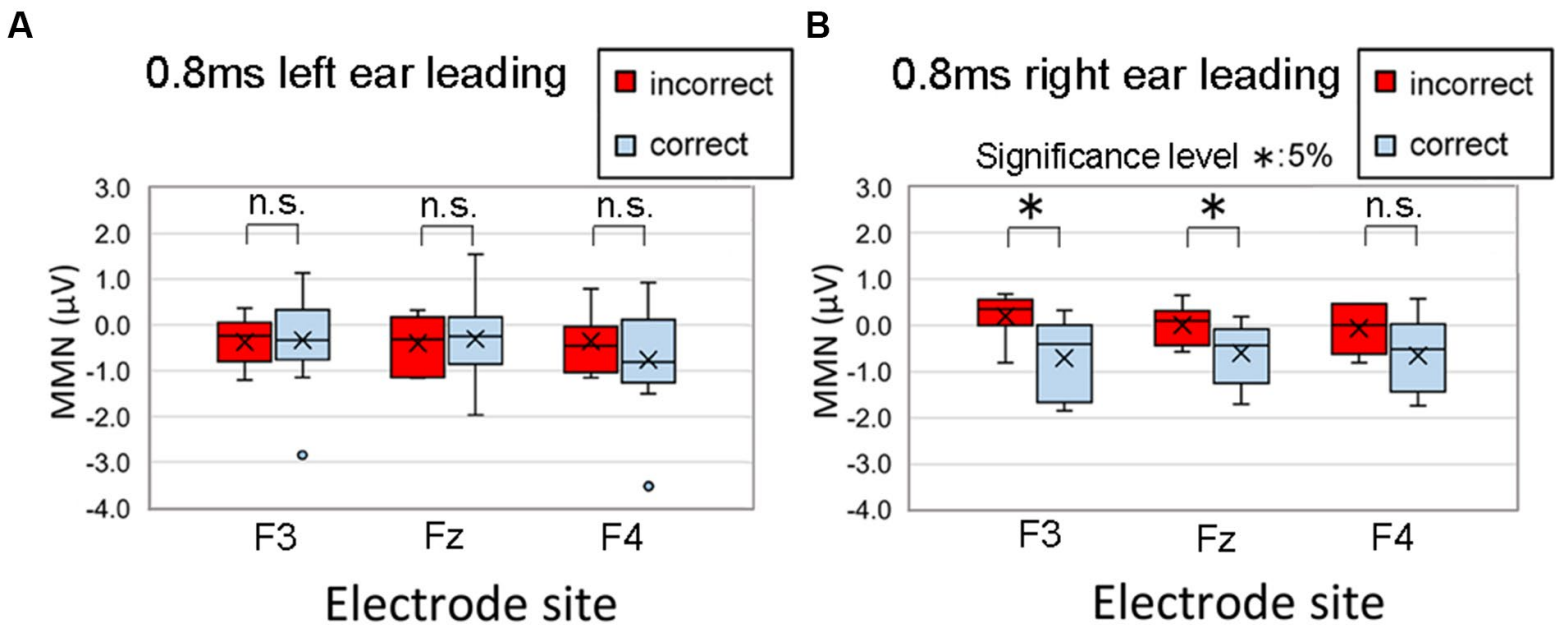
Comparison of the MMNs at F3, Fz, and F4 of 19 older adult participants with incorrect (red box) and correct (blue box) lateralization responses. A correct judgment was defined as a lateralization to the side of the leading ear in the onset part. In the figure, **(A)** “Right” responses by seven participants to a sound with an ITD of 0.8 ms leading in the left ear were considered incorrect, while “Left” responses by 12 participants were considered correct; **(B)** seven participants incorrectly responded “Left” and 12 correctly responded “Right.” The legends for the boxplot are the same as those in Figure 7. Significant differences in MMN were recognized at F3 (*p* = 0.019) and Fz (*p* = 0.041) in the right ear-leading condition. The MMN values did not significantly differ between correct and incorrect responses for the electrode sites in the left ear-leading condition. Reproduced from Morita et al. (2023a) with the permission of the Acoustical Society of America. The figure was slightly modified to describe symbols indicating significant differences between correct and incorrect responses. MMN, mismatch negativity.

Here, we recapitulate the MMN results for the condition with an ITD of 0.8 ms for the older adult participants in the cited study, which showed a characteristic trend. Figure 12, which is a reproduction of Figure 8 in the study by Morita et al. (2023a), depicts MMNs at the F3, Fz, and F4 electrode sites in the frontal region for older adult participants at an ITD of 0.8 ms. A correct judgment was defined as a lateralization to the side of the leading ear in the onset part in the lateralization test. In Figure 12A, the “Right” responses of seven participants to a sound with an ITD of 0.8 ms leading in the left ear were considered incorrect, while the “Left” responses of 12 participants were considered correct. In Figure 12B, seven participants incorrectly responded “Left” and 12 correctly responded “Right.” Notably, MMN values with greater negativity indicated heightened brain activity, whereas values approaching zero implied a comparable brain response to standard stimuli.

Herein, we provide a direct quotation of our past study, showing the statistical results of a two-factor ANOVA with the between-subjects factor of incorrect/correct responses (two levels) and the within-subject factors of electrode site (F3, Fz, and F4; three levels):

In the case of the left ear leading condition, no significant difference was observed between incorrect and correct responses [*F*(1,17) = 0.048, *p* = 0.828, effect size *f* = 0.050] or electrode site [*F*(2,34) = 1.889, *p* = 0.167, effect size *f* = 0.143], and no significant interaction was noted [*F*(2,34) = 2.583, *p* = 0.090, effect size *f* = 0.132]. Conversely, the ANOVA results for the right ear leading condition differed, revealing a significant main effect of incorrect/correct responses [*F*(1, 17) = 5.466, *p* = 0.032, effect size *f* = 0.527, power (1 − β) = 0.974]. There was no significant difference according to electrode site [*F*(2, 34) = 0.643, *p* = 0.532, effect size *f* = 0.042], nor was a significant interaction observed [*F*(2, 34) = 1.720, *p* = 0.194, effect size *f* = 0.105; Morita et al., 2023a].

We retested the data to calculate multiple comparisons of the MMN values between correct and incorrect responses using Bonferroni correction. Significant differences were recognized at F3 (*p* = 0.019) and Fz (*p* = 0.041) in the right ear-leading condition. The MMN values did not significantly differ between correct and incorrect responses for the electrode sites in the left ear-leading condition. These findings suggest a correlation between lateralization performance and brain activity, indicating that proficiency in lateralization performance is associated not only with peripheral function but also with central and/or corpus callosum function (Morita et al., 2023a).

### 4.3. Implications

This study provides clear evidence of the REA in perceiving pure tones among young participants, but not among older adult participants. The functional loss of central-auditory processes in the older adult population can be attributed to a combination of cognitive ability decline and reduced efficiency in interhemispheric transfer of information (Martin and Jerger, 2005). While our study does not identify the specific brain regions involved in auditory information processing among the older adult participants, it does highlight a significant reduction in sensitivity to input in the right ear in this population compared with the young population. Therefore, to enhance auditory information processing in older adults and enable them to perform at levels similar to those of young adults, it is crucial to prioritize right ear support in hearing assistive technologies, as pointed by Walden and Walden (2005).

These implications underscore the importance of considering age-related changes in auditory processing and tailoring assistive technologies to meet the specific requirements of older adults. These tailored considerations may ultimately enhance their quality of life and social engagement. Further research exploring the underlying neural mechanisms and developing targeted interventions can contribute to the advancement of hearing assistive technology for older adults.

### 4.4. Conclusion

This study aimed to examine the horizontal lateralization ability of older adult participants in comparison to young participants by administering 1-kHz pure tones with ITDs to the left and right ears. The authors investigated the impact of four distinct rise times (2, 5, 20, and 50 ms) on the onset portions and observed that both older adult and young participants exhibited diminished recognition of the onset portion as the rise time increased. Moreover, the study demonstrated that the traditional theory of REA, commonly utilized in language recognition, is applicable not only to language but also to the sound lateralization of pure tones with ITD among young participants. Young participants performed better in both left-and right-ear inputs when the rise time was short, whereas a pronounced REA was observed when the rise time was extended. However, the REA phenomenon was not observed in older adult participants.

The influences of hearing threshold and preferred ear on sound lateralization performance were found to be relatively insignificant. Thus, factors other than the hearing threshold or preferred ear contribute to REA in young participants or its decline with age. Furthermore, one hypothesis postulates that the decline in central nervous system function in aging individuals, potentially attributed to the deterioration of the left hemisphere and/or corpus callosum function, may also contribute to this phenomenon.

This study aimed to comprehend the auditory processing characteristics of the aging population by examining their performance on lateralization tasks. To further clarify their auditory characteristics, investigations on the brain regions related to these characteristics are essential for future research.

### 4.5. Limitations

First, although this study clearly demonstrated that the left–right ear asymmetry differed between the young and older adult samples, the samples were restricted to these age groups. We suggest for future researchers to explore other age groups, such as infants or middle-aged populations, as their evidence may provide new insights into the characteristics of auditory processing across different age groups.

Second, this experiment was contained to the use of 1-kHz pure tones. Using other sounds, such as narrow band noise, in future research may yield other, different, and interesting outcomes.

Third, the findings pertaining to the asymmetry between the left and right ears in this study were based on the subjective responses provided by the participants. To obtain a more comprehensive understanding of auditory information processing in older individuals, it is essential to conduct further investigations that specifically aim to elucidate the precise brain regions involved in this process.

## Data availability statement

The original contributions presented in the study are included in the article/supplementary material, further inquiries can be directed to the corresponding author.

## Ethics statement

The studies involving humans were approved by Ethics Committee of the Chuo University. The studies were conducted in accordance with the local legislation and institutional requirements. The participants provided their written informed consent to participate in this study.

## Author contributions

KM was involved in research design, data collection, analysis, and manuscript preparation. YG was used for data collection and analysis. TT was involved in the project and research design. All authors contributed to the article and approved the submitted version.

## Funding

This study was supported by funding from the Institute of Science and Engineering of Chuo University and JSPS KAKENHI (grant number 22K12948).

## Conflict of interest

The authors declare that the research was conducted in the absence of any commercial or financial relationships that could be construed as a potential conflict of interest.

## Publisher’s note

All claims expressed in this article are solely those of the authors and do not necessarily represent those of their affiliated organizations, or those of the publisher, the editors and the reviewers. Any product that may be evaluated in this article, or claim that may be made by its manufacturer, is not guaranteed or endorsed by the publisher.

## Abbreviations

HL: Hearing level
ITD: Interaural time difference
MMN: Mismatch negativity
NN: Neural network
REA: Right ear advantage
